# Clinical features and outcomes of seven patients with COVID-19 in a family cluster

**DOI:** 10.1186/s12879-020-05364-1

**Published:** 2020-09-03

**Authors:** Yiling Zhang, Cheng Zhang, Ying Hu, Hongmei Yao, Xianchun Zeng, Changrong Hu, Li Zhao, Xiangyan Zhang, Xianwei Ye

**Affiliations:** 1 Department of Respiratory and Critical Care Medicine, Guizhou Provincial People’s Hospital, State Key Laboratory of Diagnosis and Treatment of Lung Immune Diseases, Guiyang, 550002 China; 2 Department of Respiratory Medicine, Panzhou People’s Hospital, Liupanshui, China

**Keywords:** Clinical features, SARS-CoV-2, COVID-19,family cluster, Case report

## Abstract

**Background:**

The family cluster is one of most important modes of severe acute respiratory syndrome coronavirus 2 (SARS-CoV-2) transmission throughout China, and more details are needed about how family clusters cause the spread of coronavirus disease 2019 (COVID-19).

**Case presentation:**

We retrospectively reviewed 7 confirmed cases from one family cluster. Both clinical features and laboratory examination results were described. Patient 1 had been in close contact with someone who was later confirmed to have COVID-19 in Wuhan City before he returned back to his hometown. He had dinner with 6 other members in his family. All the persons developed COVID-19 successively except for one older woman who neither had dinner with them nor shared a sleeping room with her husband. Six patients had mild or moderate COVID-19 but one older man with underlying diseases progressed into the severe type. After general and symptomatic treatments, all the patients recovered.

**Conclusions:**

In a family cluster, having dinner together may be an important mode for the transmission of SARS-CoV-2. In this setting, most cases are mild with a favorable prognosis, while elderly patients with underlying diseases may progress into the severe type. For someone who has close contact with a confirmed case, 14-day isolation is necessary to contain virus transmission.

## Background

In December 2019, an outbreak of a novel coronavirus disease was reported in Wuhan, China. The virus was subsequently renamed SARS-CoV-2, which caused COVID-19. At present, we know that SARS-CoV-2 infects ciliated bronchial epithelial cells and type-II pneumocytes through angiotensin-converting enzyme 2 (ACE2) as receptor [1, 2]. Its infection leads to higher mortality [3–6], which is a serious threat to global public health. The sustained human-to-human transmission of SARS-CoV-2 in China is mostly caused by family clusters and intimate contact, but the details of the former transmission mode have not yet been fully investigated. Therefore, we introduce a family cluster of 7 patients with COVID-19 in Liupanshui City, Guizhou Province, and analyze their exposure to sources of transmission, symptoms, coexisting disorders, disease severity, radiologic findings, clinical characteristics, laboratory findings and treatment. Our findings will facilitate the understanding of the clinical features of COVID-19 in family clusters.

## Results

There were 8 members in one family, and 7 developed COVID-19. Patient 1 was the only person who had contact with a confirmed patient in Wuhan, but he had no history of exposure to the Huanan seafood market. On January 17, 2020, he returned home and lived with other members in one household, and he had dinner with 6 of them. Except for Patient 1, the others had neither been to Wuhan nor had contact with other confirmed cases since last year. Patient 1 shared a sleeping area with his wife (Patient 2), as did Patient 5 with Patient 6 (Patient 5’s wife). Patient 1’s mother did not like to share a bedroom with her husband (Patient 4) because he went to the toilet at night. In addition, the mother cooked in their kitchen and did not like to have dinner with the other family members (the family did not disclose the reason). Therefore, she was the only one in the family who did not develop COVID-19.

One patient had underlying diseases including bronchiectasis, latent tuberculosis and postoperative esophageal cancer, and 1 patient had pneumoconiosis. The common symptoms were cough, expectoration, sore throat or myalgia and fatigue. No patients developed severe respiratory failure or multiple organ dysfunction. Seven patients were discharged, and no patients died (Tables 1 and 2). All patients showed normal serum levels of C-reactive protein (CRP), creatine kinase, troponin, bilirubin, creatine isoenzyme and D-dimer. One patient (case 4) showed lymphopenia, elevated procalcitonin and BNP, decreased OI, and anemia (Tables 3 and 4). Four patients showed abnormalities on chest CT scan, which revealed ground-glass opacities or patchy opacities in different areas of the unilateral or bilateral lungs. All patients received antiviral treatment and traditional Chinese medicine. Two patients received antibiotic treatment.

**Table 1:** Personal information and clinical characteristics of 7 patients with COVID-19

Characteristics	case1(patient1)	case2(patient2)	case3(patient3)	case4(patien4)	case5(patient5)	case6(patient6)	case7(patient7)
Age (years)	49	38	10	75	43	41	18
Sex	male	female	male	male	male	female	female
Hospitalization days	16	12	6	38	9	8	14
Time from illness onset to first hospital admission (days)	7	6	9	4	6	7	6
Disease severity	common	mild	mild	severe	common	common	mild
Coexisting disorders	–	–	–	Bronchiectasis Obsolete tuberculosis Postoperative esophageal cancer	Pneumoconiosis	–	–
Smoking histrory	–	–	–	20 years>400pcs/year	20 years>400pcs/year	–	–
Drinking history	–	–	–	10 years,200 ml/Day	–	–	–
Treatment							
Antibiotic treatment	–	Moxifloxacin	–	Moxifloxacin Meropenem Piperacillin - tazobactam sodium Fluconazole	–	–	–
Antiviral therapy	Interferon alpha inhalation Lopinavir/ritonavir	Interferon alpha inhalation;Lopinavir/ritonavir	Interferon alpha inhalation;Lopinavir/ritonavir	Interferon alpha inhalation Lopinavir/ritonavir Arbidol Chloroquine phosphate	Interferon alpha inhalation Lopinavir/ritonavir	Interferon alpha inhalation Lopinavir/ritonavir; Arbidol	Interferon alpha inhalation Lopinavir/ritonavir
Chinese patent medicine treatment	Traditional Chinese medicine	Traditional Chinese medicine Tanreqing Xiyanping	Traditional Chinese medicine	Traditional Chinese medicine	Traditional Chinese medicine	Traditional Chinese medicine	Traditional Chinese medicine
Oxygen therapy							
Air	yes	yes	yes	no	yes	yes	yes
Nasal catheter	–	–	–	2 L/min	–	–	–
High flow oxygen	–	–	–	–	–	–	–
Non-invasive ventilator	–	–	–	–	–	–	–
Invasive ventilator	–	–	–	–	–	–	–
Days of immune booster use	1 week	–	–	2 week	–	–	–
Convalescent plasma therapy	–	–	–	Intravenous infusion of convalescent plasma 5 times, totaling 1000 ml	–	–	–
Nucleic acid recovery positive	yes	–	–	–	–	–	–
Admission to intensive care unit	–	–	–	Admission on day 28 and discharge on day 35	–	–	–
Anxiety, depression	Psychological counseling	–	–	Psychological counseling	–	–	–
Discharge from hospital							
Death	–	–	–	–	–	–	–
Recovery	yes	yes	yes	yes	yes	yes	yes

**Table 2:** The symptoms of 7 patients with COVID-19

Symptoms	case1	case2	case3	case4	case5	case6	case7
Cough	yes	yes	–	yes	–	–	–
Sputum production	yes	yes	–	yes	–	–	–
Fever	yes	–	–	–	yes	–	–
Sore throat	–	–	yes	–	–	yes	–
Fatigue	yes	–	–	–	–	–	–
Headache	–	–	–	–	–	–	–
Diarrhea	–	–	–	–	–	–	–
Stomach ache	–	–	–	–	–	–	–
Bloating	–	–	–	–	–	–	–
Nausea	–	–	–	–	–	–	–
Vomit	–	–	–	–	–	–	–
Palpitations	–	–	–	–	–	–	–
Chest tightness	–	–	–	yes	yes	–	–
Shortness of breath	–	–	–	yes	–	–	–
Nasal congestion	–	–	–	–	–	–	–
Myalgia or arthralgia	–	–	–	–	–	–	–

**Table 3:** The laboratory findings of 7 patients with COVID-19 (Admission)

Variable	case1	case2	case3	case4	case5	case6	case7
PH (7.35–7.45)	7.32	7.4	7.375	7.386	7.352	7.369	7.333
PO_2_ (80-100 mmHg)	90.1	108	77.3	63.2	73.5	87.3	92.6
PCO_2_ (35-45 mmHg)	40.4	41	41.4	34.9	44.4	43.8	97.2
OI (400-500 mmHg)	310	372	368	253	350	415	440
White blood cell count (4–10 × 10^9^/L)	5.93	9.12	6.13	9.11	7.74	5.98	5.11
Neutrophil percentage (45–77%)	52.6	81.3	60.4	84.6	47.4	52.9	54.7
Lymphocyte count (0.8–4× 10^9^/L)	2.34	1.23	1.91	0.29	3.35	2.25	1.73
Haemoglobin (131-172 g/L)	163	132	129	85	164	150	172
Eosinophil percentage (0.5–5%)	1.6	0.3	0.8	0.6	1.5	4	2.4
Platelet count (100–300× 10^9^/ L)	318	159	204	211	283	323	265
ERS (0-20 mm /h)	46	29	6	89	41	64	28
CRP (0-8 mg/L)	2.84	2.24	4	12.31	16	15	22
Procalcitonin (≤0.5(ng/ml)	0.01	0.032	0.041	1.34	0.022	< 0.02	0.041
GLU (3.9–6.1 mmol/L)	5.1	7.2	4.7	6.8	4.4	5.3	4.2
Alanine aminotransferase (8- 40 U/L)	27	18	6	17	9	20	7
Aspartate aminotransferase (5 -40 U/L)	22	32	17	18	23	18	19
Urea nitrogen (2.9–8.2 mmol /L)	4.7	3.5	3.7	5.6	6.2	3.1	2.9
Creatinine (40-106umol/L)	75.2	52.5	42.5	65	85.7	50.8	57.6
Glutamyl transpeptidase (8- 58 U/L)	41	19	12	18	22	18	14
Lactate dehydrogenase (115 -220 U/L)	192	94	203	184	226	170	181
Total bilirubin (5.1–20 umol /L)	6.6	6.4	5.7	5.4	5.7	5.8	21.5
Direct bilirubin (0–6.8umol/ L)	0.2	5	1	0.6	2.4	2	7.3
Indirect bilirubin (2-17umol/ L)	6.4	1.4	4.7	4.8	3.3	3.8	14.2
Myoglobin (<21 ng/ml)	12	18	<21	20	<21	<21	<21
Creatine kinase (25-196 U/ L)	76	10	81	28	87	44	37
Creatinase isoenzyme (0–26 U/L)	7	21	6	22	16	15	7
Troponin (0–0.1 ng/ml)	0.01	0.001	<0.003	0.012	0.005	<0.003	0.003
APTT (26-44 s)	37.3	32.8	32.4	34.9	35.5	35.1	35.2
D-dimer (0-1 mg/ml)	0.4	0.47	0.43	0.52	0.43	0.44	0.45
BNP (<300 pg/ml)	–	–	–	3143	–	–	–

**Table 4:** The laboratory findings of 7 patients with COVID-19 (Discharge)

Variable	case1	case2	case3	case4	case5	case6	case7
Laboratory findings							
PH(7.35–7.45)	7.37	7.389	7.401	7.394	7.395	7.379	7.349
PO_2_(80-100 mmHg)	83.1	112	97.3	85	80.5	80.1	94.1
PCO_2_(35-45 mmHg)	38.2	37.1	38	39	40.5	46.3	41.4
OI(400-500 mmHg)	395	386	463	340	383	381	448
White blood cell count (4–10 × 10^9^/L)	5.33	5.7	3.65	7.8	6.07	4.71	4.42
Neutrophil percentage (45–77%)	54.4	70.4	38.6	78.7	49.3	62	46.6
Lymphocyte count (0.8–4 × 10^9^/L)	1.9	1.33	1.87	0.94	2.47	1.26	1.84
Haemoglobin (131-172 g /L)	151	130	138	89	169	132	142
Eosinophil percentage (0.5–5%)	1.6	0.4	1.7	2.2	0.6	4.71	6.7
Platelet count (100–300 × 109/L)	247	200	208	273	236	273	242
ERS (0–20 mm/h)	12	32	10	82	21	20	5
CRP (0-8 mg /L)	1.23	1.12	4	16	1.12	0.74	4
Procalcitonin (≤0.5(ng/ml)	0.037	< 0.02	0.032	0.17	0.035	< 0.02	0.02
GLU(3.9–6.1 mmol/L)	4.9	5.5	5.2	7.74	4.9	4.8	4.1
Alanine aminotransferase (8-40 U/L)	25	4	7	8	18	13	10
Aspartate aminotransferase (5-40 U/L)	14	9	18	12	24	14	9
Urea nitrogen (2.9–8.2 m mol/L)	4	2.2	13	8.94	5.2	3	1.9
Creatinine (40-106umol/L)	87.3	81.2	3.7	58.1	85.8	55	54.1
Glutamyl transpeptidase (8-58 U/L)	34	12	50.6	18	22	17	16
Lactate dehydrogenase (115-220 U/L)	155	117	177	180	171	114	162
Total bilirubin (5.1-20umol /L)	12.6	3.6	6.2	6	4	3.7	6.5
Direct bilirubin (0–6.8 u mol/L)	1.7	1.3	1.6	1.2	2	1.2	1.1
Indirect bilirubin (2–17 umol/L)	10.9	2.3	4.6	4.8	2	2.5	5.4
Myoglobin (<21 ng/ml)	28.99	< 21	< 21	< 21	23.49	< 21	< 21
Creatine kinase (25–196 U/L)	119	32	67	39	28	23	30
Creatinase isoenzyme (0- 26 U/L)	6	3	4	26	7	2	8
Troponin (0–0.1 ng/ml)	0.037	< 0.003	0.003	< 0.001	0.006	< 0.003	< 0.003
APTT (26-44 s)	36	31.4	36	35.7	30.7	31.5	33.3
D-dimer (0-1 mg/ml)	0.89	0.47	0.3	2.73	0.43	0.44	0.45
BNP<300 pg/ml)	–	–	–	196	–	–	–

On discharge, all the abnormal indexes above were greatly improved.

## Case presentation

### Case 1

**Fig. 1:**
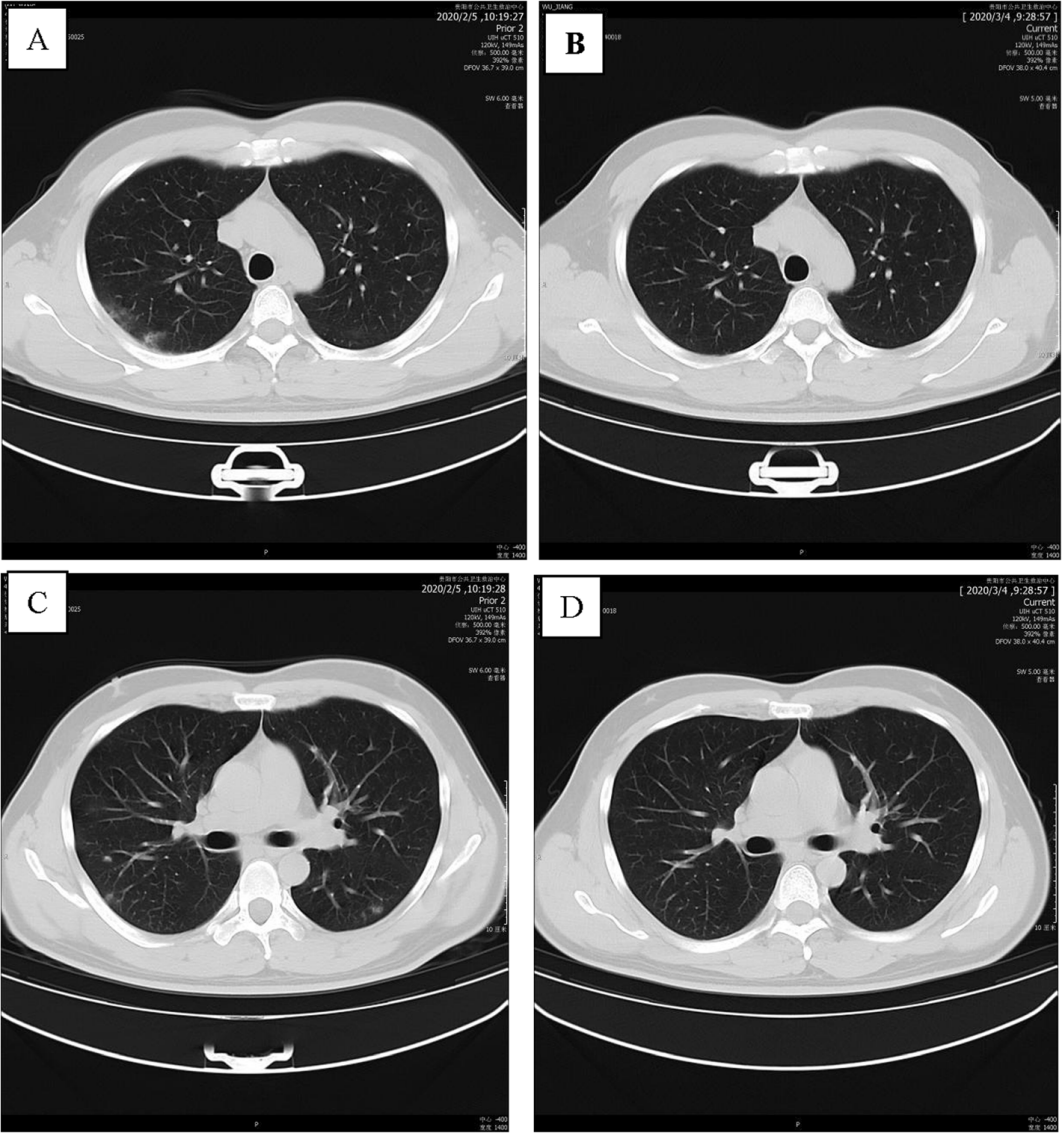
Chest CT images of A 40-year-old man with COVID-19 infection. **a, c** Transverse chest CT images showed scattered Ground-glass opacities exudation in both lungs on admission. **b, d** Transverse chest CT images showed no abnormalities in both lungs on discharge

A 40-year-old man with a long history of residence in the epidemic area of Wuhan. On January 16, 2020, he had dinner in Wuhan with an individual who was later confirmed to have COVID-19. He returned to his hometown, Panzhou, Guizhou Province, by car on January 17, 2020. He was admitted to a fever clinic at the local hospital on January 28, 2020, with symptoms of fever (the first symptom), cough, sputum production and fatigue. His throat swab samples were immediately collected and real-time reverse transcription polymerase chain reaction (rRT-PCR) assay confirmed that he had developed COVID-19. Chest CT revealed scattered ground-glass opacities and exudation in both lungs (Fig. 1a, c). Therefore, according to the New Coronavirus Infection Pneumonia Protocol published by the National Health Commission of China, the patient was diagnosed by the local hospital, with a moderate case [7], and treated with inhaled interferon alpha (10 million international units [IUs] daily),oral lopinavir/ritonavir (800/200 mg daily) and traditional Chinese medicine (recipes were adjusted based on different patient situations) for 5 days. After 3 days, the patient had no symptoms. After that, he was admitted to Guizhou Provincial Jiangjun Mountain Hospital on February 4, 2020. The treatment plan was the same as that of the local hospital. After 11 days, all his symptoms had disappeared. On February 15th and 17th, 2020, his throat swab samples tested negative for SARS-CoV-2 antigens by two rRT-PCR tests, taken more than 24 h apart. Reexamination of chest CT showed no abnormalities, and he was discharged from the hospital for 14 days of medical isolation and observation in Guizhou Workers’ Hospital near Guizhou Provincial Jiangjun Mountain Hospital. There, he did not receive any treatment at all. On day 14 of medical isolation, the throat swab tested positive again for SARS-CoV-2 antigens, and he was admitted to the hospital on March 2 and discharged on March 8 again after antiviral treatment. He had no symptoms during this period, and no abnormality was found on chest CT (Fig. 1b, d).

### Case 2

**Fig. 2:**
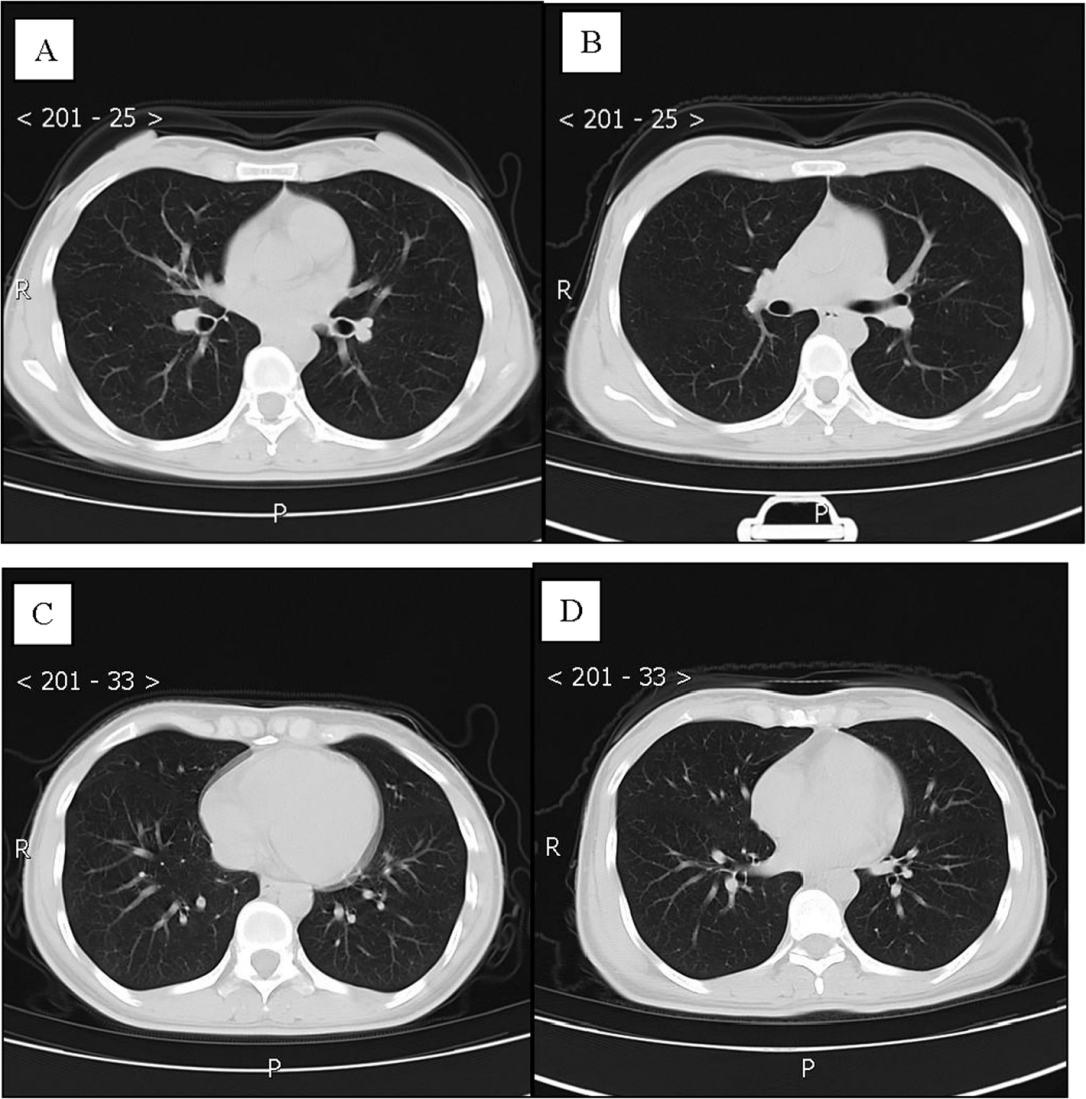
Chest CT images of A 38-year-old woman with COVID-19 infection. **a, c** Transverse chest CT images showed no abnormalities in both lungs on admission. **b, d** Transverse chest CT images showed no abnormalities in both lungs on discharge

Patient 2, a 38-year-old woman, was the wife of patient 1 and had a history of close contact (such as having dinner) with patient 1. She was admitted to a fever clinic at the local hospital on January 29, 2020, with symptoms of cough (the first symptom) and sputum production. Her throat swab samples were immediately collected and rRT-PCR assay confirmed that the patient had COVID-19. A chest CT scan revealed no abnormalities (Fig. 2a, c). Therefore, the patient was diagnosed by the local hospital, with a mild case of COVID-19 [7]. She was immediately admitted to the isolation ward and treated with inhaled interferon alpha, oral lopinavir/ritonavir and traditional Chinese medicine for antiviral therapy. Since she had sputum production, moxifloxacin was given orally to prevent bacterial infections. After 5 days, on February 5, 2020, the patient was admitted to Guizhou Provincial Jiangjun Mountain Hospital. The treatment plan was the same as that of the local hospital. On February 14th and 16th, 2020, her throat swabs, taken more than 24 h apart, tested negative for SARS-CoV-2 antigens by two rRT-PCR assays. She had no symptoms and was discharged from the hospital for 14 days of medical isolation observation in the hospital above. On day 14, her throat swab sample was negative again. No abnormality was found on chest CT (Fig. 2b, d).

### Case 3

**Fig. 3:**
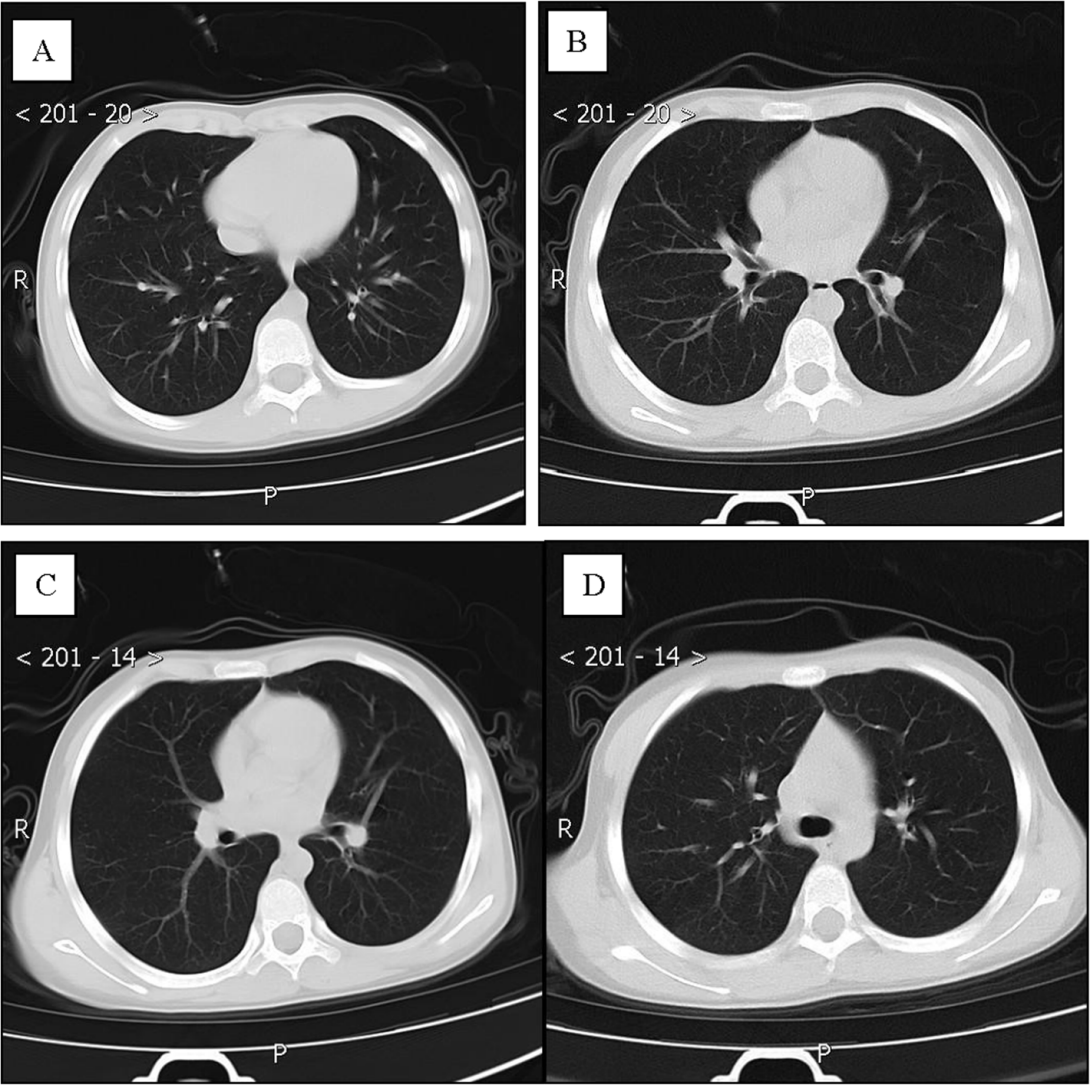
Chest CT images of A 10-year-old boy with COVID-19 infection. **a**, **c** Transverse chest CT images showed no abnormalities in both lungs on admission. **b**, **d** Transverse chest CT images showed no abnormalities in both lungs on discharge

Patient 3, a 10-year-old boy, was the son of patient 1 and had a history of close contact (such as having dinner) with patient 1. He was admitted to a fever clinic at the local hospital on January 29, 2020, with symptoms of sore throat. His throat swab samples were immediately collected and were tested by rRT-PCR assay, and the patient was diagnosed with COVID-19. A chest CT scan revealed no abnormalities (Fig. 3a, c). Therefore, he was diagnosed with a mild case of COVID-19 [7] and then immediately admitted to the isolation ward and treated with inhaled interferon alpha, oral lopinavir/ritonavir and traditional Chinese medicine for antiviral therapy. After 5 days, on February 5, 2020, the patient was admitted to Guizhou Provincial Jiangjun Mountain Hospital. The treatment plan was the same as that of the local hospital. On February 8th and 10th, 2020, his throat swabs tested negative for SARS-CoV-2 antigens by two rRT-PCR assays, more than 24 h apart. At that time, his symptoms had also disappeared. Therefore, he was discharged from the hospital for 14 days of medical isolation observation in the hospital above. On day 14, his throat swab tested negative again. No abnormality was found on the chest CT (Fig. 3b, d).

### Case 4

**Fig. 4:**
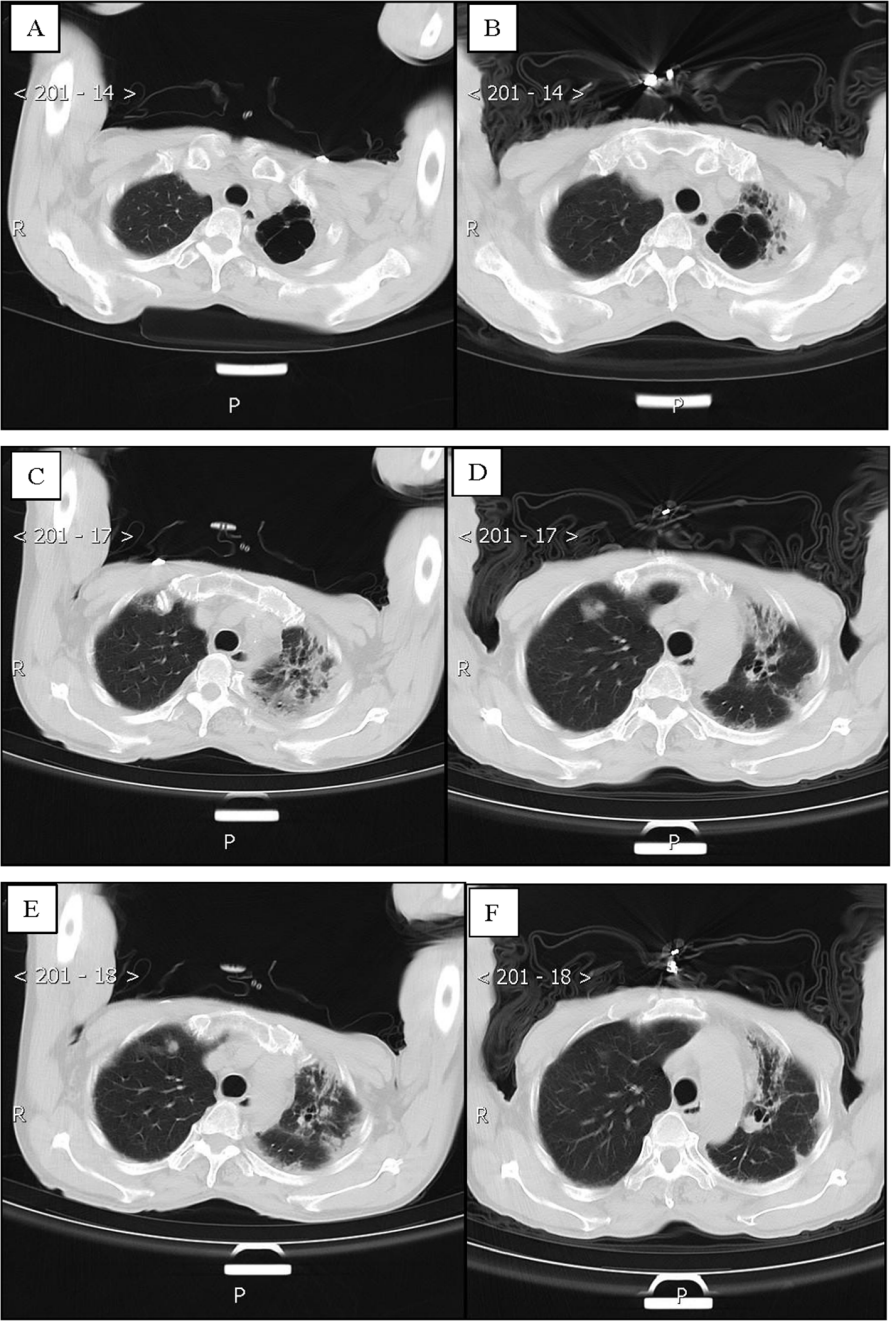
Chest CT images of A 75-year-old man with COVID-19 infection and bronchiectasis, obsolete tuberculosis. **a**, **c**, **e** Transverse chest CT images revealed extensive patchy exuding shadows and fibre stripe in left lung, and damage changes of upper lobe or left lung on admission. **b**, **d**, **f** Transverse chest CT images showed patchy exuding shadows mostly was absorbed in left lung on discharge

Patient 4, a 75-year-old man, was the father of patient 1 and had a history of close contact (such as having dinner) with Patient 1. He was admitted to a fever clinic at the local hospital on January 28, 2020, with symptoms of cough (the first symptom), sputum production, chest tightness and shortness of breath. He was complicated by coexisting diseases: postoperative esophageal cancer, bronchiectasis, latent tuberculosis, hypoxemia and anemia. He had a more than 10-year history of smoking and a 20-year history of drinking, and he had quit both for more than 10 years. His throat swab samples were immediately collected and confirmed by rRT-PCR assay. The patient was diagnosed with COVID-19. Chest CT on admission revealed extensive patchy opacities and reticulation in the left lung and changes indicating damage in the upper lobe of the left lung (Fig. 4a, c). Therefore, the patient was diagnosed with a severe case of COVID-19 pneumonia [7] and was immediately admitted to the isolation ward and received supplemental oxygen through a nasal cannula. His oxygenation index ranged from 240 to 250. He was treated with inhaled interferon alpha, oral lopinavir/ritonavir and traditional Chinese medicine for antiviral therapy. Moxifloxacin and piperacillin-tazobactam were given intravenously to prevent bacterial infections. After 5 days, the patient was admitted to Guizhou Provincial Jiangjun Mountain Hospital on February 4, 2020. Laboratory examination showed that his hemoglobin (85 g/L) and lymphocyte counts (0.29× 10^9^/L) were decreased, while BNP (3143 pg/ml) was significantly increased.

The treatment plan was different from that of the local hospital. He was successively given inhaled interferon alpha; lopinavir/ritonavir, Arbidol, and chloroquine phosphate as antiviral therapy; and meropenem, piperacillin-tazobactam sodium and fluconazole to prevent secondary infection. However, laboratory test results of this patient showed a progressive decline in the absolute number of lymphocytes, a delay in viral clearance, no obvious absorption on chest CT, and a progressive rise in procalcitonin. He was admitted to the intensive care unit (ICU) on the 28th day and discharged from the ICU on the 35th day of hospitalization. Intravenous infusion of convalescent plasma was administered 5 times, totaling 1000 ml, to attenuate lung inflammation. Fortunately, the treatment was very effective and successful: his symptoms were alleviated, with an increase in OI and lymphocyte count and a decrease in BNP. On March 9th and 11th, 2020, his throat swab samples were examined again and were negative for SARS-CoV-2 antigens by two rRT-PCR tests, taken more than 24 h apart. Reexamination of chest CT showed significant absorption of extensive patchy opacities in the left lung (Fig. 4b, d). Therefore, he was discharged from the hospital for 14 days of medical isolation observation in the hospital above.

### Case 5

**Fig. 5:**
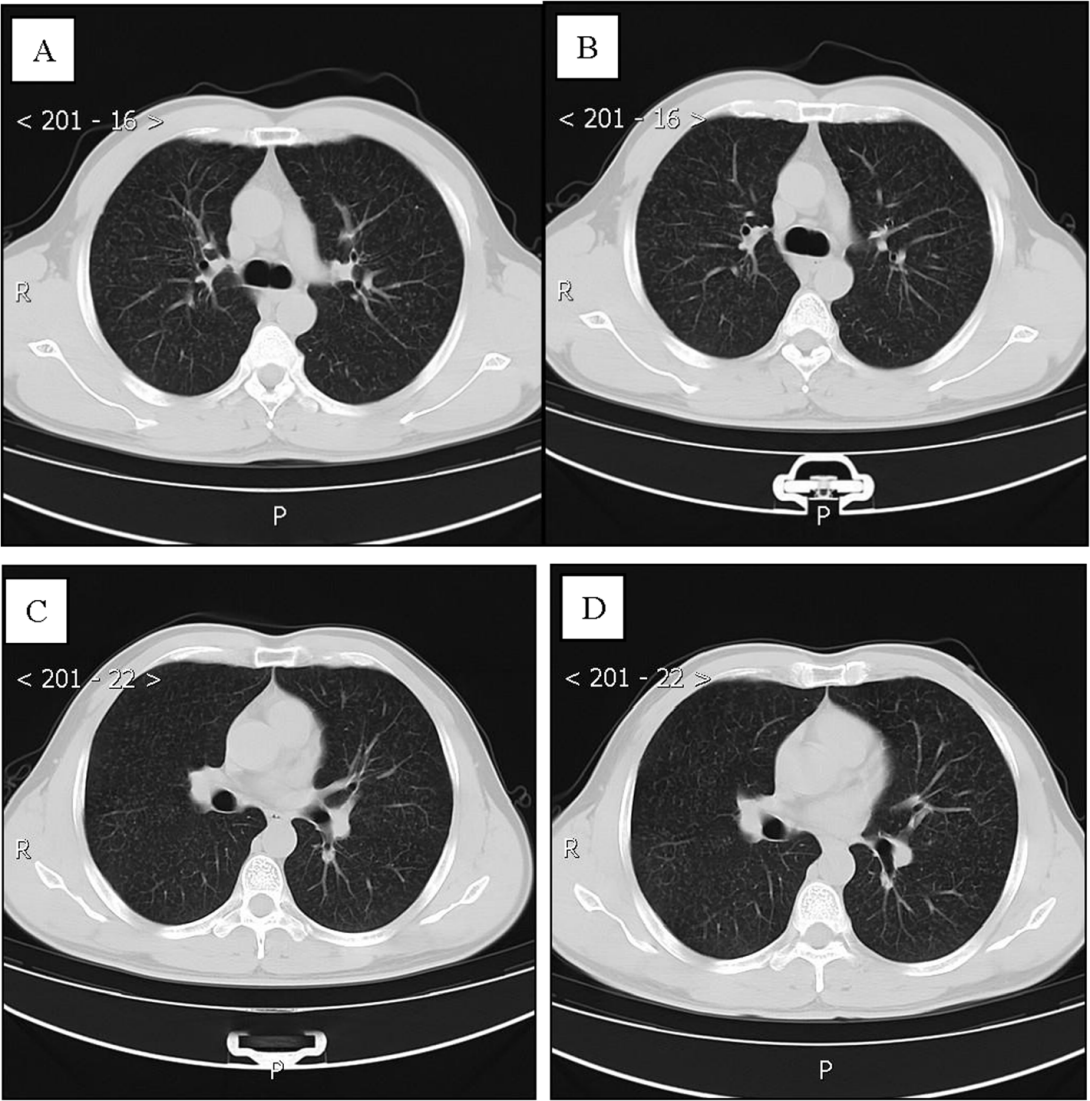
Chest CT images of A 43-year-old man with COVID-19 infection and pneumoconiosis. **a**, **c** Transverse chest CT images showed diffuse multiple nodules in both lungs on admission. **b**, **d** Transverse chest CT images showed diffuse multiple nodules in both lungs on discharge

Patient 5, a 43-year-old man, was the brother of patient 1 and had a history of close contact (such as having dinner) with patient 1. He was admitted to a fever clinic at the local hospital on January 28, 2020, with symptoms of chest tightness (the first symptom), low-grade fever, night sweats, and anorexia. He had a coexisting disorder, pneumoconiosis and a history of smoking for more than 20 years. His throat swab samples were collected and tested positive by rRT-PCR assay. Chest CT revealed diffuse small nodules in both lungs (Fig. 5a, c). Laboratory findings of the patient showed no evidence to support a tuberculosis diagnosis. Therefore, the patient was diagnosed by the local hospital, with a mild case of COVID-19 [7]. He was treated with inhaled interferon alpha, oral lopinavir/ritonavir and traditional Chinese medicine for antiviral therapy. After 4 days, on February 4, 2020, the patient was admitted to Guizhou Provincial Jiangjun Mountain Hospital. The treatment plan was different from that of the local hospital.

After some time, his symptoms disappeared. On February 10th and 12th, 2020, two throat swab samples taken more than 24 h apart tested negative for SARS-CoV-2 antigens by two rRT-PCR assays. Reexamination of chest CT showed no significant changes (Fig. 5b, d). Therefore, he was discharged from the hospital for 14 days of medical isolation observation in the hospital above. Similarly, on day 14, his throat swab samples tested negative for SARS-CoV-2 antigens by rRT-PCR. He had no symptoms during this period.

### Case 6

**Fig. 6:**
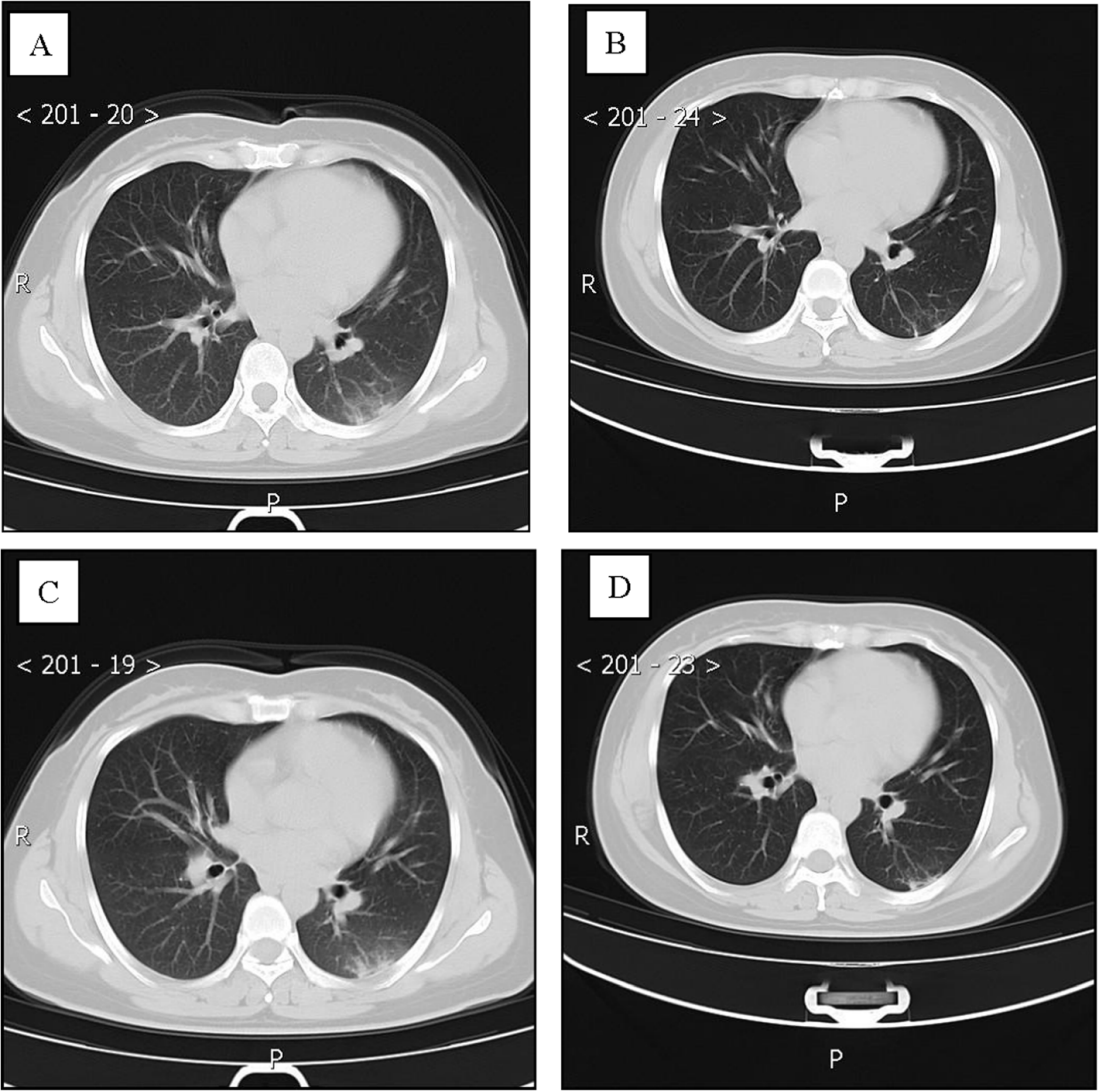
Chest CT images of A 41-year-old woman with COVID-19 infection. **a**, **c** Transverse chest CT images showed ground-glass opacity changes in the left lower lung on admission. **b**, **d** Transverse chest CT images showed tremendously absorption of ground-glass exudate in the left lower lung on discharge

Patient 6, a 41-year-older woman, was the sister-in-law of patient 1 and had a history of close contact (such as having dinner) with patient 1. She was admitted to a fever clinic at the local hospital on January 29, 2020, with a sore throat. Her throat swab samples were immediately collected and rRT-PCR assay confirmed that the patient had COVID-19. Chest CT scan revealed ground-glass opacities in the left lower lung on admission (Fig. 6a, c). The patient was diagnosed by the local hospital with a moderate case of COVID-19 pneumonia [7], and she was then treated with inhaled interferon alpha, oral lopinavir/ritonavir and traditional Chinese medicine for antiviral therapy. After 7 days, on February 7, 2020, the patient was admitted to Guizhou Provincial Jiangjun Mountain Hospital. The treatment plan was the same as that of the local hospital. After a few days, her symptoms disappeared. On February 12th and 14th, 2020, two throat swab samples taken more than 24 h apart tested negative for SARS-CoV-2 antigens by rRT-PCR. Her chest CT revealed that the ground-glass opacities in the lower left lung were mostly absorbed (Fig. 6b, d). Therefore, she was discharged from the hospital for 14 days of medical isolation observation in the hospital above. Similarly, her throat swab samples tested negative for SARS-CoV-2 by rRT-PCR on day 14, and she had no symptoms during the isolation period.

### Case 7

**Fig. 7:**
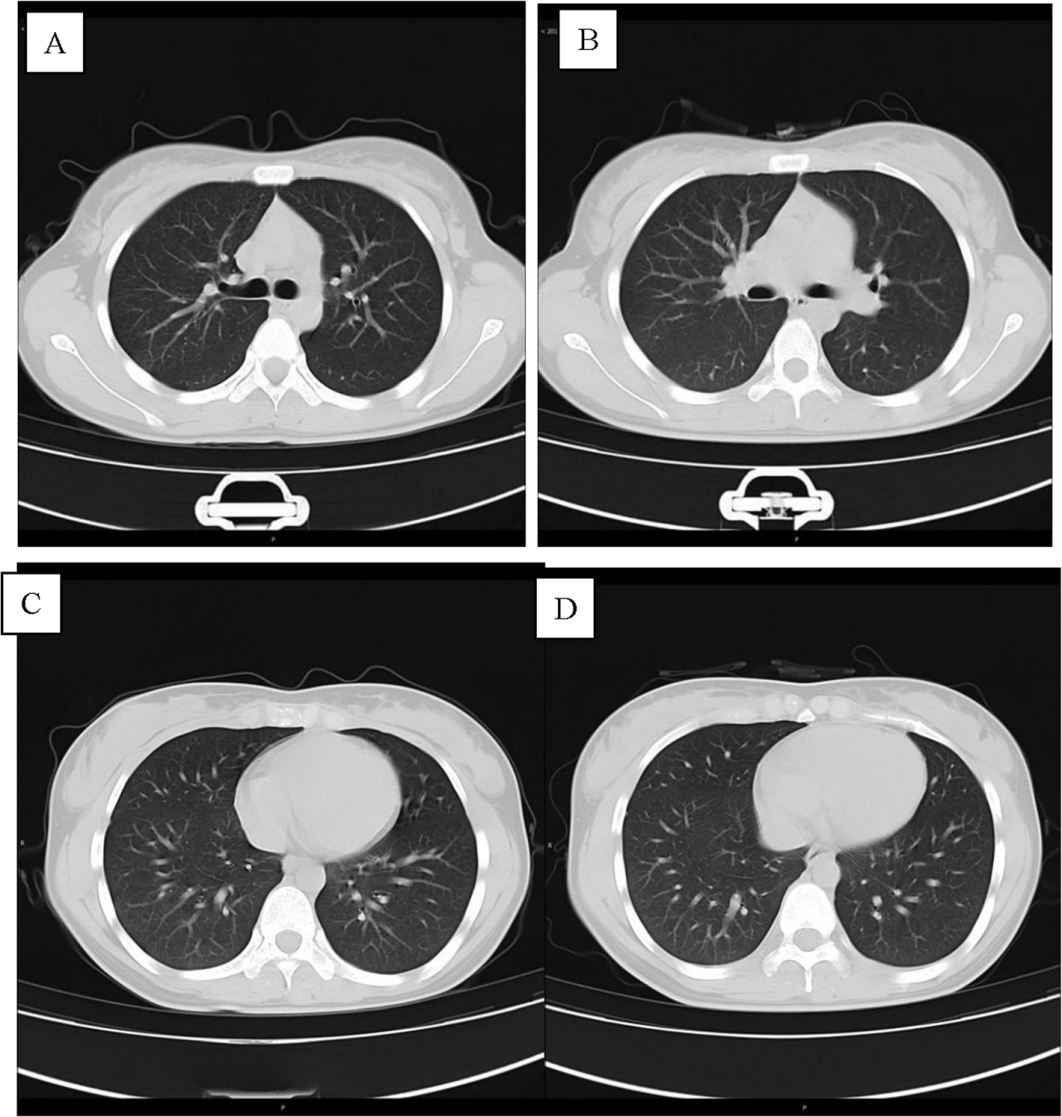
Chest CT images of A 18-year-old girl with COVID-19 infection. **a**, **c** Transverse chest CT images showed no abnormalities in both lung on admission. **b**, **d** Transverse chest CT images showed no abnormalities in both lungs on discharge

Patient 7, an 18-year-old girl, was the niece of patient 1 and had a history of close contact with patient 1 (such as having dinner). On January 29, 2020, she was admitted to a fever clinic at the local hospital without any symptoms. Her throat swab samples were immediately collected and confirmed by rRT-PCR assay, and the patient was diagnosed with COVID-19. A chest CT scan showed no abnormalities (Fig. 7a, c). Therefore, she was diagnosed in the local hospital with a mild case of COVID-19 [7] and was immediately admitted to the isolation ward and treated with inhaled interferon alpha, oral lopinavir/ritonavir and traditional Chinese medicine for antiviral therapy. After 5 days, on February 5, 2020, the patient was admitted to Guizhou Provincial Jiangjun Mountain Hospital. The treatment plan was the same as that of the local hospital. On February 15th and 17th, 2020, two throat swab samples taken more than 24 h apart tested negative for SARS-CoV-2 antigens by two rRT-PCR assays. On day 14 of medical isolation, her throat swab samples again were negative. Therefore, she was discharged from the hospital, and she had no symptoms during the period (Fig. 7b, d).

## Discussion and conclusions

In this study, 7 patients were included, with slightly different clinical features and outcomes. Of the patients, patient 1 had dinner with a confirmed patient in Wuhan, so he was the first person who developed COVID-19 in his family. At home, he had close family contact with his father, wife, son, elder brother, sister-in law, and niece. For example, he had dinner with other family members and talked to them at close range. Therefore, this may be the main reason why the other 6 persons were infected with SARS-CoV-2.

As we know, there were 8 persons living in this household, but only patient 1’s mother did not develop COVID-19, although she was of an older age, which attracted our attention. Therefore, we performed a further investigation and found that she had seldom had close contact with other family members since patient 1 returned back from Wuhan. For undisclosed reasons, she neither had dinner with the others nor shared a sleeping room with her husband (patient 4), so she was not infected. Based on the evidence above, we think having dinner may be an important mode for viral transmission among family members. Whether sharing a sleeping room, is another factor that easily causes transmission is not yet clear. More studies are needed to find the answer.

Since patients 2–6 had no close contact with other confirmed cases except for patient 1, we speculate that they may have been infected by patient 1 through close contact. Unlike those in Wuhan [3], most of the patients had mild illness with common symptoms such as fever, cough, expectoration, sore throat, and chest tightness, although one older man (patient 4) had more severe disease. According to the Chinese protocol, clinical classifications of COVID-19 are as follows: (1) Mild cases: The clinical symptoms are mild, and no pneumonia manifestations can be found in imaging. (2) Moderate cases: Patients have symptoms such as fever and respiratory tract symptoms, and pneumonia manifestation can be seen in imaging. (3) Severe cases: patients meeting any of the following: 1) respiratory distress, indicated by a RR ≥30 breaths/min; 2) pulse oxygen saturation (SpO2) ≤ 93% on room air at a resting state; or 3) arterial partial pressure of oxygen (PaO2)/oxygen concentration (FiO2) ≤300 mmHg. Patients with > 50% lesion progression within 24 to 48 h in pulmonary imaging should be treated as having severe disease.

Although the 7 patients lived together, they had different clinical manifestations, different incubation periods and different outcomes. Two patients were asymptomatic, 3 patients had no abnormalities on chest CT scan, 2 patients showed some ground glass opacities on the chest CT, and 1 patient had multiple diffuse nodules in both lungs on chest CT, indicating pneumoconiosis. Unlike the children, the elderly patient suffered from coexisting diseases such as bronchiectasis, postoperative malignant tumor, and latent tuberculosis and had more severe symptoms with hypoxemia, anemia, lymphopenia, heart failure and so on. His chest CT imaging manifestations were more serious, his viral clearance time was delayed, and the treatment time was prolonged, so he was admitted to the ICU for 7 days, which significantly increased the complexity of treatment. We think old age, underlying diseases and lymphopenia may contribute more to the progression of the disease. During hospitalization, he was given convalescent plasma, blood transfusion, immune enhancers, traditional Chinese medicine, oxygen therapy, combined anti-infection medications, and quadruple anti-viral treatment. Based on the Chinese protocol, he was regarded as having severe disease, while the rest in this family were regarded as having mild or moderate disease.

Family clustering is currently one of the most common modes of transmission of COVID-19 [5, 8–10]. In this study, we described the details of this disease in a family cluster and learned that in family settings, the clinical features, outcomes, and prognosis of infection may be affected by the patient’s age, coexisting diseases, lymphocyte count, and so on. Although there are currently no specific medicines or vaccines for COVID-19, an individualized treatment plan according to the patient’s clinical characteristics and laboratory tests is needed as soon as possible.

To our knowledge, early isolation, early diagnosis and early management may be the most effective ways to reduce the incidence of COVID-19 in China. Therefore, it is important to know the epidemiological characteristics and clinical features of patients. This study showed that all the patients had no history of exposure to the Huanan seafood market in Wuhan, patient 1 had close contact with someone with confirmed COVID-19, while the remaining 6 patients had only close contact with him by family gathering, which suggests that this disease has a strong infectious ability. Personal protective measures (especially keeping a proper distance from others) are strongly recommended. In addition, we also found that lymphopenia, anemia and some coexisting diseases are common features in patients with COVID-19 and might be the key factors related to disease severity [11–13]. Our findings will facilitate understanding of the clinical features and provide new insights into family clusters of COVID-19 patients.

According to the cases presented above, we conclude that in a family cluster, having dinner may be an important mode for SARS-CoV-2 transmission. In this setting, most of the cases were mild with favorable prognosis, but elderly patients with underlying diseases may progress to severe disease. For someone who has close contact with a confirmed case, a 14-day isolation period is necessary to contain virus transmission in a family.

## Supplementary information

**Additional file 1.**

## Data Availability

The data related to this study are mostly included in this article. For more details, please contact the first author, Yiling Zhang.

## Abbreviations

SARS-CoV-2: Severe acute respiratory syndrome coronavirus 2
COVID-19: Coronavirus disease 2019
rRT-PCR: Real-time reverse transcription polymerase chain reaction
CT: Computed tomography
CRP: C-reactive protein
OI: Oxygenation index
ESR: Erythrocyte sedimentation rate
BNP: Brain natriuretic peptide
ICU: Intensive care unit
