# Supplementary material for: Clinical features and outcomes of seven patients with COVID-19 in a family cluster
Source: BMC Infect Dis. 2020 Sep 3;20:647. doi: 10.1186/s12879-020-05364-1 (PMC7468093; doi:10.1186/s12879-020-05364-1)
Supplement: Supplementary file 1 — Additional file 1. [file 12879_2020_5364_MOESM1_ESM.doc]

Grandfather(Case 4)

Grandmother(Healthy )

Father (Case 1)

Mother(Case 2)

Uncle(Case 5)

Son(Case 3)

Aunt(Case 6)

Daughter(Case 7)

Family tree
